# Human cortical folding across regions within individual brains follows universal scaling law

**DOI:** 10.1038/s42003-019-0421-7

**Published:** 2019-05-20

**Authors:** Yujiang Wang, Joe Necus, Luis Peraza Rodriguez, Peter Neal Taylor, Bruno Mota

**Affiliations:** 1 Interdisciplinary Computing and Complex BioSystems (ICOS), School of Computing, Newcastle University, Newcastle upon Tyne, NE4 5TG UK; 2 Institute of Neuroscience, Newcastle University, Newcastle upon Tyne, NE1 7RU UK; 3 Institute of Neurology, University College London, London, WC1N 3BG UK; 4 Instituto de Física, Universidade Federal do Rio de Janeiro, Av. Athos da Silveira Ramos, 149 - Cidade Universitaria, Rio de Janeiro, 21941-909 Brazil

**Keywords:** Scale invariance, Brain, Alzheimer's disease, Power law, Biophysical models

## Abstract

Different cortical regions vary systematically in their morphology. Here we investigate if the scaling law of cortical morphology, which was previously demonstrated across both human subjects and mammalian species, still holds within a single cortex across different brain regions. By topologically correcting for regional curvature, we could analyse how different morphological parameters co-vary within single cortices. We show in over 1500 healthy individuals that, despite their morphological diversity, regions of the same cortex obey the same universal scaling law, and age morphologically at similar rates. In Alzheimer’s disease, we observe a premature ageing in the morphological parameters that was nevertheless consistent with the scaling law. The premature ageing effect was most dramatic in the temporal lobe. Thus, while morphology can vary substantially across cortical regions, subjects, and species, it always does so in accordance with a common scaling law, suggesting that the underlying processes driving cortical gyrification are universal.

## Abstract (web summary)

Yujiang Wang et al. show that despite regional differences in brain morphology, different regions in individual brains still obey the universal scaling law of cortical morphology. Although premature aging was observed in Alzheimer's disease patients, these changes also followed the scaling law, suggesting that cortical morphology is guided by universal principles.

## Introduction

Cortical folding, or gyrification, is one of the most striking morphological features that can appear in mammalian brains, with many recent studies investigating its mechanisms^1–7^. Recently, we proposed a universal law that relates average thickness *T*, exposed area *A*_*e*_ and total area *A*_*t*_ of a cerebral cortex, and describes its degree of folding:

1 $$A_t\sqrt T = kA_e^\alpha ,$$

where our model predicts *α* = 5/4. This model is based on the assumption that cortical morphology minimises an effective free energy that takes into account a pressure-like term (hypothesised to be exerted by white matter axonal tension and hydrostatic cerebral spinal fluid pressure) and the self-avoiding nature of the cortical surface^8^.

The conceptual significance of this scaling law is that variations in e.g. the exposed area, must be compensated by countervailing changes in thickness or total area. We have observed and reported this in action, first across cortices of different species^8^, then across different human individuals^9^. In spite of a great morphological variety in both studies, the quantities co-vary such that the same universal scaling relation, with the same values for *α* = 1.25, is approximately followed in all cases. The only free parameter is thus *k*, or offset, a dimensionless coefficient that is related to the pressure term. Examining the human data in greater detail, we have shown that there is no significant variation in the scaling exponent *α,* but we noted a systematic decrease of the offset parameter *k* with age^9^, which may be interpreted as a slackening of white matter axonal tension or a decrease in white matter axonal density. We can think of both the exponent *α* and the offset *k* as two natural probes into cortical morphology, applicable across any number of conditions and groups. The former tests the universality of the gyrification mechanism, and the latter measures changes in physical properties.

As currently formulated however, these probes are only applicable in group analyses across either species or individuals, but not across regions within a single cortex. It is known, however, that there are systematic variations in cortical morphology between cortical regions and region-specific changes with age (e.g. refs. ^10–14^). Furthermore, there are a number of neurological conditions that have been observed to produce morphological anomalies primarily or solely in specific cortical regions (e.g. refs. ^15–18^). Hence, natural questions are: Are the mechanisms of gyrification the same for all cortical regions within a single cortex? Are there systematic regional differences in the timing and extent of folding during ageing, and in health vs. disease? The main purpose of this paper is to generalise our morphological probes to cortical regions within a single cortex so as to be able to answer these questions.

To answer the questions above, two main methodological steps must be taken: The first is to find a consistent way of partitioning a cortex. The largest natural partition divides each hemisphere into four lobes (frontal, occipital, parietal, and temporal), and the definition of each is tolerably consistent across both individuals and species^19,20^. Smaller partitions are also possible, e.g. using predefined atlases of specific regions of interest. The second step is to assess if each partition separately follows the same universal scaling law (Eq. (1)) within a single cortex.

One might think that for the second step, simply performing the same scaling analysis on each region as was done for the whole cortex would be sufficient. However, a simple thought experiment shows the problem: Let us assume a uniform cortex with constant cortical thickness *T* is partitioned into segments having the same gyrification index (*g* = *A*_*t*_/*A*_*e*_). Then the exposed *A*_*e*_ and total *A*_*t*_ areas of each partition would correspond to the same fraction of their respective values for the full cortex. Thus, in the *x* = log(*A*_*e*_) and $y = \log (A_t\sqrt T )$ projection, the data points corresponding to each partition would all line up along a line of slope 1 (signifying constant gyrification index), ordered according to size. A heterogeneous cortex would introduce further dispersion perpendicular to the line of slope 1 (see a more detailed description in Supplementary Note 1 in ref. ^21^). In either case, this approach would not capture the scaling law, but only differences in partition size, thickness, and gyrification index.

Instead we propose to rely on the known invariance of the integrated Gaussian curvature on a closed surface. (This result is a consequence of the famous Gauss–Bonnet theorem^22^, which states that the total integrated Gaussian curvature in a closed surface—such as that of cortical hemisphere to a close approximation—is a topological invariant.) For each partition, we obtain equivalent *A*_*e*_′ and *A*_*t*_′ values that a putative full cortical hemisphere would have, if it had on average the same gyrification index, cortical thickness and average Gaussian curvature for its total and exposed surfaces as the partition. These estimates ($A_e^\prime$ and $A_t^\prime$) can then be used for Eq. (1) to evaluate if the scaling law also applies across partitions of the same cortex.

Here, we investigate the scaling law over different brain regions of the brain, first in three independent cohorts of healthy subjects, and describe the effect of ageing on the scaling law. We then apply the analysis to a cohort of Alzheimer’s disease (AD) patients and show that we can detect a ‘premature ageing’ effect in morphological terms in those patients, particularly in the temporal lobe. Finally, we also discuss some limitations of our study and thoughts for future investigations.

## Results

### Lobes of the same cortex also obey the universal scaling law

**Fig. 1:**
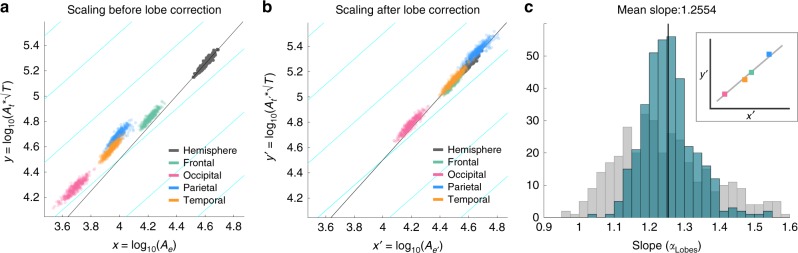
Scaling behaviour for different lobes of the same cortex. **a** Scaling behaviour is shown for raw data of lobes in different colours, and for the whole hemisphere in grey. Grey line shows the linear regression for the whole hemisphere (slope = 1.2557). Cyan lines indicate contour lines of constant gyrification index in this space (slope = 1) along which our correction term operates. **b** Same as **a**, only with correction term applied to the lobes to reconstruct their equivalent whole hemisphere data points. **c** Histogram of subject-specific regression slopes of the lobes is shown in blue. Solid black line indicates the mean slope across subjects (being 1.2554). Grey histogram shows the subject-specific regression slopes of smaller subdivisions of a single cortex. Inset schematically exemplifies how the slope for a specific hemisphere was obtained through regression across its four lobes. For all panels, the HCP data was used, and we only included data for age 22–25 in this figure

Figure 1a shows how cortical lobes naturally disaggregate according to their size and thickness in a *x* = log(*A*_*e*_) and $y = \log (A_t\sqrt T )$ plot. As expected, the lobes separately do not follow the universal relation (Eq. (1)) for gyrification. All lobes are shifted to the bottom left in this plot compared to the whole hemisphere, and the shift is more pronounced for smaller lobes (e.g. occipital lobe) due to larger decreases in surface areas.

To arrive at measures that can be used to evaluate the scaling law of different lobes in the same cortex, we derived estimates of $A_e^\prime$ and $A_t^\prime$ by applying a multiplicative correction to the areas *A*_*e*_ and *A*_*t*_ of the lobes (see Supplementary Note 1 in ref. ^21^ for details). We obtained the estimates of *A*_*e*_′ and *A*_*t*_′ of each lobe, and left the average thickness of the lobe *T* unaltered. These quantities can now be compared directly to each other by plotting *x*′ = log_10_($A_e^\prime$) against $y{\prime} = \log _{10}(A_t^\prime \sqrt T )$, and to the original, whole hemisphere (Fig. 1b).

In Fig. 1b, it is visually clear that the correction maps the data for all lobes (coloured data points) very close to the universal scaling behaviour of the whole hemisphere (grey data points and grey line). Note that the correction moves the data points along the direction of constant gyrification index (cyan lines), as the correction term preserves gyrification index. Note also that the corrected areas for all lobes now cluster closer to the original full cortex areas. In some cases, notably in the parietal/occipital lobe, the corrected areas can be larger/smaller than that of the whole hemisphere, which is a consequence of different lobes having gyrification indexes that are either lower or higher than the whole cortical average.

When we restrict our analysis only to the four lobes belonging to each hemisphere, it becomes clear that, even when different lobes are dissimilar, they also follow the same scaling rule as the whole hemispheres after their morphological parameters are corrected appropriately. In Fig. 1c, a distribution of slopes is obtained for the sets of four lobes in each hemisphere separately. Note that this derives a scaling exponent for each hemisphere separately (denoted as *α*_Lobes_), and is no longer a group-based estimate across different cortices (we denote the group-based estimate of slope as *α*_Hemispheres_). Also note that there were no significant differences between males and females (*p* = 0.0645 in a two-sided ranksum test), hence we present all our data by grouping both together. The average slope across the group is 1.2554, i.e. very close to the predicted value of 1.25. In Supplementary Note 2^21^, we also performed this analysis using a linear mixed effect model, effectively estimating a joint slope across different cortices. The results show that the 95% confidence bounds around the group mean of the slope are ±0.01, and that the subjects do not have significantly different slopes to each other (*p* = 0.52).

So far we investigated the scaling relationship between different lobes of the same cortex. But our topological method can in principle also be applied to different or more detailed partitions of the cortex. We thus extended our analysis to the full set of partitions given by the Desikan–Killiany atlas. We found that partitions located on the medial wall, and very small partitions, were not given adequate correction terms due to their Gaussian curvature being near zero. The full details can be found in Supplementary Note 3 in ref. ^21^. However, after removing those problematic regions, we obtain a distribution of slope that has a very similar mean (1.244) as that obtained for the lobes, but with a larger variance (Fig. 1c, grey histogram). This indicates that our method can be applied to smaller partitions of the cortex, but with some limitations. We will discuss these in more detail later and in Supplementary Note 3 in ref. ^21^.

### Slope of lobes does not change over age

**Fig. 2:**
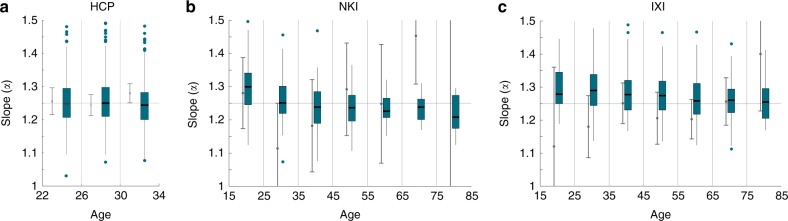
Individual hemisphere slope estimates over age vs. group slope estimates. **a** Distribution of slope estimates (*α*_Lobes_) from lobe-based regression is shown as box plots (dark green) over different age groups for the HCP data. For comparison, the group slope estimate (*α*_Hemispheres_) is shown as light grey error bars (95% CI). **b**, **c** Same as in **a**, but for the NKI and IXI data, which span a wider age range. Supplementary Note 4 in ref. ^21^ shows the raw data underlying this plot

To test if the individual slopes (*α*_Lobes_) change over age, we repeated the analysis from Fig. 1 for different age ranges (Fig. 2). To enable comparison to the group slope estimate (*α*_Hemispheres_) we also used age categories. For the HCP dataset, there were enough subjects to allow for a 4-year age categorisation. For all other datasets 10-year categories were used. In all cases, the distribution of individual slopes *α*_Lobes_ cluster around 1.25 within one standard deviation. The raw data with continuous age is shown in Supplementary Note 4 in ref. ^21^. There is, however, a drift of slope values with age, where a steeper slope is observed for younger subjects, and a shallower slope is seen for older subjects. This drift is significant for all three datasets in a linear regression (*p* < 0.05 for all datasets), see details in Supplementary Note 4 in ref. ^21^. We return to this observation in Discussion. It is also worth noting that *α*_Lobes_ estimates generally align better with 1.25 than the slope estimates based on the full cortex (*α*_Hemispheres_, grey lines in Fig. 2, which are the same slope estimates as in our previous publication^9^). This better alignment can be quantified by measuring the distance of the mean slope to 1.25 in each age category; in each dataset this distance is lower on average across age categories. This is not surprising, given that the group slope estimates are directly influenced by outliers caused by e.g. segmentation and surface reconstruction errors^9^. In lobe-based estimates, however, such outliers have little effect on the median of the distribution.

### Offset for all lobes decrease with age

**Fig. 3:**
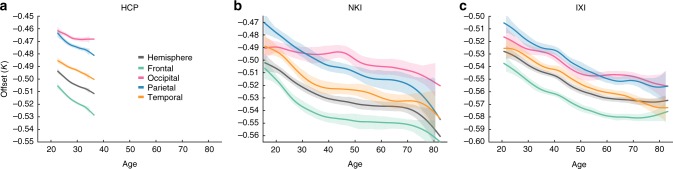
Offset changes over age for different lobes and the whole hemisphere. **a** Offset (*K*_Lobe_) is shown to change over age for different lobes for the HCP (900 subject release) data in four different colours. Note that shaded areas indicate 95% bootstrapped confidence intervals for the mean, not the variance. For comparison, the offset for the whole hemisphere (*K*_Hemisphere_) is shown in black. **b**, **c** Same as in **a**, but for the NKI and IXI data, which span a wider age range. Supplementary Note 4 in ref. ^21^ shows the raw data underlying this plot

Based on the previous result, we assume a slope *α*_Lobes_ for each cortex of 1.25 across all ages in all datasets. This allows us to calculate the offset $K_{\mathrm{Lobe}} = \log (k\prime ) = \log (A_t^\prime \sqrt T ) - \frac{5}{4}\log (A_e^\prime)$ separately for each lobe and each cortex. Figure 3 shows the offset *K*_Lobe_ over age for all four lobes, and also the entire hemisphere (where $K_{\mathrm{Hemisphere}} = \log (k) = \log (A_t\sqrt T ) - \frac{5}{4}\log (A_e)$) for reference. Overall, the offset decreases with age for all lobes, as expected from our results for the hemisphere^9^. Generally, the lobes show the same rate of decrease as the hemisphere, although there is a hint of the occipital lobe decreasing at a slightly slower speed. Indeed when testing the slope of the decrease (*K* ~ *age*) for differences between the lobes, significant differences were found (Supplementary Note 5 in ref. ^21^).

We additionally note small systematic differences in offset between the lobes and between different datasets. We return to this observation in Discussion.

### Scaling of lobes in Alzheimer’s disease is not altered

**Fig. 4:**
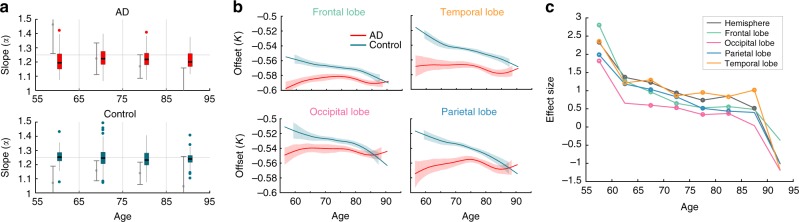
Slope and offset over age for Alzheimer’s subjects. **a** Slope estimates based on different lobes of the same hemisphere (box plots) for Alzheimer’s patients (AD) and controls. As a reference the horizontal grey line indicates the predicted 1.25 slope. To enable comparison, we also show the group-based slope estimates as grey error bars. **b** Offset (assuming 1.25 as slope) is shown for AD and control groups for different lobes. Solid line indicates the mean, and shaded area shows the 95% bootstrapped confidence intervals for the mean. **c** Effect size of offset (*K*_Lobe_) from the control vs. AD between-group comparisons at different lobes. Empty circles show where the effect was significant (*p* < 0.05) under a two-sided ranksum test

To enable further comparison of our results here with our previous results on the whole cortex scaling, we also investigate the same Alzheimer’s dataset we analysed in ref. ^9^. The dataset is a cross-sectional cohort of about 200 Alzheimer’s patients and 200 healthy controls in the same age range. In terms of the slope of the scaling law, we previously found that a significant gender-specific change may occur in AD compared to controls. We re-investigated this by deriving a distribution of slopes from how the lobes scale within the same cortex. Figure 4a shows that the previously found difference in *α*_Hemispheres_ disappears with this estimation of slope based on the lobes (*α*_Lobes_).

We also previously noted that even the slopes in the control cohort seemed to be decreasing, which also is not the case with our lobe-based analysis. All control slope distributions are centred on 1.25, without any significant changes with age (Fig. 4a).

In terms of offset (calculated again assuming a slope of 1.25), we observe the same trend for the lobes as for the whole hemisphere: *K*_Lobe_ for the AD group remains low and roughly stationary for all ages, while the control group slowly drifts down to this level over age (Fig. 4b). When applying a linear regression to test if *K*_Lobe_ changes with age, all lobes show a non-significant (*p* > 0.05) slope with age for AD, but significant (*p* < 0.05) for controls.

There are, however, some subtle but significant differences between the lobes. When measuring the difference between AD and control group (as effect size between the two distributions), the occipital/temporal lobe is shown to have the smallest/biggest effect size overall (Fig. 4c). The effect sizes also show a sharp decrease overall after age 60, then stays roughly constant until age 85.

## Discussion

In summary, we demonstrated that lobes of the same cortex follow the same universal scaling law as shown in group analysis across individuals and species. With same caveats, the same also holds true for smaller partitions of a single cortex. The universal scaling of lobes is true for healthy human brains of different ages and sexes, and even holds for subjects with AD. The offset parameter, which we associated previously with axonal mechanical tension, also decreases with age in each lobe at a similar rate as for the whole cortex, except for the occipital lobe where the rate of decrease is slightly less pronounced. In AD, cross-sectionally, we observe that the difference in offset between patients and controls is largest in the temporal lobe, smallest in the occipital lobe, and stays roughly constant between age 60 and 90 for all lobes.

Our previous group analysis showed that the universal scaling law applies across different mammalian species and across human brains^8,9^. However, the same scaling behaviour within an individual cortex could not be studied until now.

In this work, we have proposed a way to extend the previous analysis to partitions of the cortex, enabling an analysis of the scaling law of an individual brain. This is a substantial step forward, as it now allows us to analyse mechanisms of folding within subjects, rather than just for a group of subjects. With this extension, we were able to show that parts of the same cortex still follow the proposed universal scaling law. Conceptually, this also confirms our hypothesis that the mechanism of folding is universal across mammalian species, within the human species, and even within a single cortex.

Furthermore, we have overcome a major methodological hurdle of analysing partitions of the same cortex: If we analysed them in the same way as we do for full cortices, they would naturally disaggregate according to the partition size, thickness, and gyrification index. Thus, no scaling law would be expected to arise from such an analysis. The methodological challenge was to reconstruct whole cortices with the same properties as the partitions, and in this way, glean the scaling properties of the reconstructed cortices. In our analysis, this was only possible through a correction term that is independent of partition size, but still represented topological size of the partition: the Gaussian curvature of the exposed area of the partitions.

Although our main analysis focused on relatively large partitions (lobes) of the cortex, we also demonstrated that the approach applies to smaller partitions (Fig. 1c and Supplementary Note 3 in ref. ^21^). In Supplementary Note 3, we show that even when using smaller partitions at the scale of gyri and sulci, our results still hold. We conclude that—as long as the Gaussian curvature of the exposed area of the partitions still contains sufficient information (i.e. it is not near zero, or dominated by noise)—then our method can be applied to study between-region scaling effects within a single cortex.

We expect our method to be particularly useful to study conditions and situations in which only part of the cortex is expected to be affected substantially. For example, many studies in the literature investigate the effects of a clinical condition on morphological parameters (e.g. cortical thickness), measured in specific cortical areas (see e.g. ref. ^23^ for a very recent review). We have shown here that the scaling parameters *k* and *α* can also be regionalised so as to be usable as powerful new local probes. We thus expect the choice of segmentation in the future to be more clinically and anatomically motivated. This will be a focus of some of our future work and, we hope, also that of others.

In summary, the main conceptual advances in this work are the new methods we created to perform a scaling analysis in a single cortex, as opposed to the group analysis shown in previous work. The individual analysis is important to make the scaling law directly useful for e.g. diagnosis, stratification, or prognosis of individual patients. The new analysis proposed here also allows us to pinpoint the spatial location of changes related to the scaling law on an individual cortex. This was previously impossible with the group analysis across subjects using full cortices.

In order to arrive at a slope estimate for each individual cortex (*α*_Lobes_), we partitioned the cortex into four different lobes, and applied a correction term to the total and exposed surface area of the lobes. The correction term is derived from the proportion of integrated Gaussian curvature located on each lobe, as a topologically invariant measure of size^22^. However, the estimation of Gaussian curvature from a surface is technically non-trivial, and numerically challenging (see Supplementary Note 1 in ref. ^21^ for details). We believe that the numerical errors (which generally increase with the size of the lobe) have led to a systematic difference in the offset of each lobe, giving bigger lobes a smaller offset, and smaller lobes a bigger offset (Fig. 3). The numerical effects may also have influenced the finding that our slope estimates seem to decrease very slightly with age (Fig. 2), as the size of the brain, and hence also the size of the lobes, is slightly smaller in older age on average^9^.

Apart from the potential numerical inaccuracies, the slight slope (*α*_Lobes_) decrease may also be driven by a genuine biological effect. It is conceivable that with a slope estimate for individual cortices, we are more sensitive to small changes with age on a more local level of the brain. In support of this, Madan et al. recently reported in a cross-sectional study a decrease in fractal dimension of the cortical ribbon with age^24^. If the scaling law is indeed true for parts of the brain, as for the whole brain, then the fractal dimension of the cortical ribbon should be 2.5, which is very close to the reported values. Both a decrease of fractal dimension with age, and similarly our decrease of slope (*α*_Lobes_) with age, may then suggest a genuine biological effect whereby the scaling behaviour may change over age.

However, a closer observation of our data shows that although there is a slight decrease in slope with age, the decrease is not consistent in absolute values across datasets. This indicates that the slight decrease is possibly caused by (technical) factors unaccounted for so far. In Supplementary Note 6 in ref. ^21^ we show that both our slope, as well as the fractal dimension are strongly correlated with our offset *K*_Hemisphere_ on an individual basis. It is difficult to draw a definite conclusion at this stage, but we speculate that rather than a genuine slope change, the observed effect here (and maybe even in ref. ^24^) is due to a secondary effect/scale that is currently assimilated in our offset parameter *K*. Future work may be able to resolve this by finding a reliable way of estimating the scaling behaviour within a cortex without being confounded by factors such as size of the cortex (or partition). Nevertheless, we can conclude from our analysis so far that lobes of the same cortex also obey the same universal scaling law as different cortices from the same species, and different mammalian species.

In terms of our scaling law, we previously interpreted a drop in *K* over age as a decrease in tension along the white matter axons^25^. However, there are other effects that would also change the value of *K* over time according to our proposed mechanism. Notably, a decrease in axonal cross-sectional density in the white matter (suggested by diffusion imaging studies^26^) would have the same effect as a proportionally equal decrease in axonal tension. Intracranial pressure changes would also affect the value of *K*. For the lobes, *K*_Lobe_ generally follows the overall behaviour of the whole cortex. Relative to each other, the frontal and temporal lobes experience the largest rate of decrease overall, and the occipital lobe appears least affected by age (Fig. 3). These results also mirror findings in the literature of cortical thickness changes over age^13,14,27,28^. The differential effect of age on cortical thinning has also been coined the ‘last in, first out’ effect. The primary sensory and motor areas develop first prenatally, and the association areas follow with a slight delay and continue growing post-natally. The association areas are earliest affected by cortical thinning in ageing, and the primary sensory and motor areas are only affected later on^27,29^. Of course, the lobes do not exactly map on to primary sensory and motor areas, but the different rates of decrease of *K* between the frontal and occipital in particular is suggestive. We conclude that our data does seem to suggest a differential effect of age on *K* (possibly caused by a decrease in white matter axonal density), where the value in the occipital lobe seems to decrease least over age.

Regarding AD, we found no significant slope (*α*_Lobes_) changes with age, or sex in either AD, or control groups, for slope estimates derived from the lobes, and no overall difference to 1.25. This finding contrasts with the results we obtained previously based on regression across a population^9^, where we detected a slope change with age in the AD group (Fig. 4a, grey error bars). We suggest that the lobe-based estimations may be more reliable and overcome some of the sample size and outlier issues in our previous work. Additionally, systematic regional variations on the cortex may have also impacted our previous results, due to the heterogeneity of cortical atrophy patterns in AD. With the lobe-based slope estimates, there might be a slight trend of a systematically shallower slope in AD than controls, although again further work is needed to show if this is a genuine characteristic of the disease or a side effect of our method. At this stage, however, we can conclude that there are no detectable slope changes in AD or controls, and all slope estimates agree with the theoretically predicted 1.25.

The changes in offset *K*_Lobe_ over age that we found in the lobes (Fig. 4b, c) agree with the overall picture we obtained previously for the whole cortex^9^. We also observed in our cross-sectional study no differential progression between the lobes in *K*_Lobe_ (and thickness, see Supplementary Note 7 in ref. ^21^) with age in AD. Morphological changes in AD are primarily observed as reductions of grey matter volume and cortical thickness^30^, which are believed to be triggered by targeted neuronal disconnections^31,32^, leading to an accelerated brain ageing process^33^. Indeed, the low and almost constant *K* in AD patients over the entire age range, agrees with these observations of accelerated ageing caused by the AD pathology. In this regard, the occipital lobe was observed to change least over age in healthy controls in all datasets, and interestingly it is also the lobe that shows least difference between AD and controls overall (Fig. 4b, c). This agrees with findings in the literature showing that AD seems to least affect the occipital lobe^30,34–36^, compared with other lobes and some of their sub-structures (e.g. middle temporal gyrus in temporal and precuneus cortex in parietal)^37,38^. We have stated previously that the consistently low value of *K* in AD, in contrast to its gradual decrease in healthy ageing, suggests that a premature morphological ageing of the cortex may be seen as an aspect of AD. On that note, it is interesting that the occipital lobe, the one least affected by AD, is also the one least affected by ageing in terms of *K*.

As for the cause of the low value of *K* in AD, it is hard to say anything definitive in view of the lack of longitudinal data to track how *K* change for individuals (and, in particular, if there is a sudden decrease of *K* at or just before the onset of AD, an effect that could have important prognostic value). However, a decrease in axonal density, something we expect would lead to a decrease in *K*, has been associated with AD in the literature (e.g. ref. ^39^).

We had previously shown^9^ that the proposed scaling law may be able to offer a new set of three natural morphological variables, that are directly and independently related to (1) cortical thickness, (2) brain size, and (3) mechanism of folding. Importantly, in this coordinate system we can disentangle the three effects from of each other, so that e.g. brain size does not influence the measure of folding (unlike the gyrification index, which is influenced by brain size). However, our previous work was limited by the fact that the scaling law could only be applied to a cohort, meaning that the folding mechanism within an individual hemisphere could not be studied.

In this work, we have proposed a way to extend these new variables to partitions of the cortex, enabling an analysis of folding of an individual brain. This is a significant step forward, as it now allows us to analyse mechanisms of folding within subjects, rather than just for a group of subjects. Conceptually, this is similar to the extension of the gyrification index to the so-called local gyrification index^40^, albeit for a complete set of independent variables rather than just one. With this extension, we were able to show that parts of the same cortex still follow the proposed universal scaling law, and hence still can be projected and understood in our natural coordinate system. Conceptually, this also confirms our hypothesis that the mechanism of folding is universal across mammalian species, within the human species, and even within a single cortex.

Future work will try and resolve the small residual dependencies on partition size we still observe, and fully develop our method into a local measure to enable its use in e.g. clinical applications for both within and between subject studies. To extend our analysis to an even smaller scale, further work needs to develop methods of analysing cortical morphology point-by-point. We are developing an extension of our cortical gyrification model along those lines, were curvature (extrinsic and intrinsic) is related to axonal incidence density and the elastoplastic mechanical properties of grey and white matter. Such new fine-grained model of course would need to be validated, ideally across the full range of interspecific/intraspecific/localised analyses that we have undertaken for the coarse-grained model that leads to the universal relation encapsulated in Eq. (1).

## Methods

### MRI and data processing

A full overview of the MRI data used is included in Supplementary Note 8 in ref. ^21^. Briefly, the datasets are obtained from four sources: The HCP data^41^ we obtained is from the 900 subjects release. For the IXI data^42^ we only used the Guy’s Hospital subdataset (site with biggest sample size). For the NKI Rockland Sample and ADNI data [Data used in preparation of this article were obtained from the Alzheimers Disease Neuroimaging Initiative (ADNI) database (adni.loni.usc.edu). As such, the investigators within the ADNI contributed to the design and implementation of ADNI and/or provided data but did not participate in analysis or writing of this report. A complete listing of ADNI investigators can be found at http://adni.loni.usc.edu/wp-content/uploads/how_to_apply/ADNI_Acknowledgement_List.pdf.], we used exactly the same subdataset as in our previous publication^9^. Informed consent was obtained from all subjects as part of the original data acquisition for the original data sources cited above.

### Partitioning of cortex

There are numerous ways to partition a cortex, and in principle the method outlined below is applicable to any choice of segmentation as long as the partitions are not too small (i.e. it must include at least some gyri and sulci). A natural initial partition, however, is dividing each hemisphere in four lobes (parietal, occipital, frontal, and temporal), the definition of each being consistent across individual humans^19^. These lobes were then further segmented into smaller regions of interest.

All partitions of the cortex were done using the Freesurfer Desikan–Killiany parcellations; for lobes we followed the Freesurfer assignments (including the assignment for the Cingulate)^43^. See Supplementary Note 1 in ref. ^21^ for more details.

### Correction term

Briefly, for all closed surfaces (or, approximately, for almost closed surfaces, such as a cortical hemisphere intersected by the corpus callosum), the integrated Gaussian (or intrinsic) curvature *I*_*G*_ is always ${\oint} GdA = 4\pi$^22^. This so-called topological invariant is thus a natural measure of the relative sizes of different partitions of a closed surface, that is insensitive to deformations and details about shape. It is also insensitive to approximating a smooth surface (such as an actual cortical surface) with a triangulated one (such as the computational representation thereof). In the latter case, the Gaussian curvature becomes concentrated on the vertices, and in each vertex it is simply the difference between 2*π* and the sum of the angles impinging on it^44^.

Now consider partitioning the cortex into sub-regions. This partition can be drawn correspondingly on the exposed surface by finding the nearest region on the folded cortex for each point on the exposed surface. The total Gaussian curvature will now be distributed among the partitions on the exposed surface. Mathematically, the Gauss–Bonnet theorem ensures that we would obtain exactly the same value using the folded surface instead of the exposed surface; in practice, using the exposed surface makes our results less sensitive to the precise placement of partition boundaries.

For reconstructing a full cortex from a partition, we require that certain properties remain unchanged: The average thickness of the reconstructed cortex *T*′^*P*^ should be the same as that of the partition *T*^*P*^. The gyrification index $g^P = \frac{{A_t^P}}{{A_e^P}}$ should also not change. Finally, the average Gaussian curvatures for the total and exposed areas in the partition, $\bar G_e^P = \frac{{I_G^P}}{{A_e^P}}$ and $\bar G_t^P = \frac{{I_G^P}}{{A_t^P}}$ should be preserved, with $I_G^P = {\int}_{A_e^P} {GdA}$ being the integrated Gaussian curvature of the partition, and $A_e^P$ the surface area of the partition. Hence:

2a $$T^{\prime P} = T^P,$$

2b $$g^{\prime P} = \frac{{A_t^{\prime P}}}{{A_e^{\prime P}}} = \frac{{A_t^P}}{{A_e^P}} = g^P,$$

2c $$\bar G_e^{\prime P} = \frac{{4\pi }}{{A_e^{\prime P}}} = \frac{{I_G^P}}{{A_e^P}} = \bar G_e^P,$$

2d $$\bar G_t^{\prime P} = \frac{{4\pi }}{{A_t^{\prime P}}} = \frac{{I_G^P}}{{A_t^P}} = \bar G_t^P.$$

From which follows the corrected total and exposed areas for the partition, now with size effects removed and directly comparable to each other and to the whole cortical hemisphere,

3 $$A_e^{\prime P} = \frac{{4\pi }}{{I_G^P}}A_e^P\,{\mathrm{and}}\,A_t^{\prime P} = \frac{{4\pi }}{{I_G^P}}A_t^P.$$

We describe the reasoning behind the correction term and estimation of $I_G^P$ in more detail in Supplementary Note 1 in ref. ^21^.

### Correction in slope estimate

To remove the systematic Gaussian error due to the dispersion in the value of $I_G^P$, let $\sigma _{\mathrm{log}I_G^P}^2 = \sigma _{I_G^P}^2/(\bar I_G^P)^2$ be the variance in the logarithm of the correction term $\mathrm{log}I_G^P$, and $\sigma ^2(\mathrm{log}A_e^{\prime P},\mathrm{log}A_t^{\prime P}\sqrt {T^P} )$ the covariance matrix for different partitions of the same hemisphere. Then it can be shown (Supplementary Note 1 in ref. ^21^) that the slope across lobes *α*_Lobes_ is given by $\alpha _{\mathrm{Lobes}} = \frac{1}{{\gamma + \sqrt {1 + \gamma ^2} }}$, where $\gamma = \frac{{\sigma _{11}^2 - \sigma _{22}^2}}{{2\sigma _{12}^2 - \sigma _{\log I_G^P}^2}}$.

### Statistics

Figure 1 included 22–25 subjects in the HCP 900 subject release data (for whom preprocessed cortical surface data existed), which resulted in 182 subjects, and thus 364 hemispheres included in the analysis. Slope estimation is described above and in more detail in Supplementary Note 1 in ref. ^21^. The sample sizes in the remaining figures are summarised as a table in Supplementary Note 8 in ref. ^21^.

Data visualisation in Figs. 2–4 was done using gramm^45^. Box plots are displayed using the function stat_boxplot (following the standard conventions: center line, median; box limits, upper and lower quartiles; whiskers, 1.5× interquartile range; points, outliers), and bootstrapped mean estimates are shown with stat_smooth(‘npoints’,np), where np is the age interval displayed in years. Grey error bars in Figs. 2 and 4a indicate the 95% confidence intervals of the whole-hemisphere slope estimate, and are the same as in our previous publication^9^ (replicated here for comparison).

In Fig. 4c, the effect size between the AD and Control was determined with Cohen’s *d* (*d* = (*m*_Control_ − *m*_AD_)/*sp*, where *m*_Control_/*s*_Control_ is the mean/standard deviation of the control cohort, *m*_AD_/*s*_AD_ is the mean/standard deviation of the AD cohort, and $sp = \sqrt {\frac{{(n_{\mathrm{Control}} - 1) \ast s_{\mathrm{Control}}^2 + (n_{\mathrm{AD}} - 1) \ast s_{\mathrm{AD}}^2}}{{(n_{\mathrm{Control}} + n_{\mathrm{AD}} - 2)}}}$). Statistical significance between the Control and AD group were determined using the two-sided ranksum test in Matlab.

The supplementary notes in ref. ^21^ further provides detailed accounts of additional statistical analyses that were not shown explicitly in the main figures.

### Reporting summary

Further information on research design is available in the Nature Research Reporting Summary linked to this article.

## Supplementary information

Reporting Summary

## Data Availability

Full data (of all surface areas, thicknesses, and curvature values in subjects) is available at zenodo (10.5281/zenodo.2595060).

## Code availability

Analysis code is available at zenodo (10.5281/zenodo.2595060).
