# Supplementary material for: Human cortical folding across regions within individual brains follows universal scaling law
Source: Commun Biol. 2019 May 20;2:191. doi: 10.1038/s42003-019-0421-7 (PMC6527703; doi:10.1038/s42003-019-0421-7)
Supplement: Supplementary file 1 — Reporting Summary [file 42003_2019_421_MOESM1_ESM.pdf]

nature research

Corresponding author(s): Yujiang Wang

Last updated by author(s): Dec 20, 2018

Reporting Summary

Nature Research wishes to improve the reproducibility of the work that we publish. This form provides structure for consistency and transparency in reporting. For further information on Nature Research policies, see Authors & Referees and the Editorial Policy Checklist.

Statistics

For all statistical analyses, confirm that the following items are present in the figure legend, table legend, main text, or Methods section.

n/a	Confirmed	
☐	☒	The exact sample size (*n*) for each experimental group/condition, given as a discrete number and unit of measurement
☐	☒	A statement on whether measurements were taken from distinct samples or whether the same sample was measured repeatedly
☐	☒	The statistical test(s) used AND whether they are one- or two-sided<br>*Only common tests should be described solely by name; describe more complex techniques in the Methods section.*
☐	☒	A description of all covariates tested
☐	☒	A description of any assumptions or corrections, such as tests of normality and adjustment for multiple comparisons
☐	☒	A full description of the statistical parameters including central tendency (e.g. means) or other basic estimates (e.g. regression coefficient) AND variation (e.g. standard deviation) or associated estimates of uncertainty (e.g. confidence intervals)
☐	☒	For null hypothesis testing, the test statistic (e.g. *F*, *t*, *r*) with confidence intervals, effect sizes, degrees of freedom and *P* value noted<br>*Give P values as exact values whenever suitable.*
☒	☐	For Bayesian analysis, information on the choice of priors and Markov chain Monte Carlo settings
☐	☒	For hierarchical and complex designs, identification of the appropriate level for tests and full reporting of outcomes
☐	☒	Estimates of effect sizes (e.g. Cohen's *d*, Pearson's *r*), indicating how they were calculated

Our web collection on statistics for biologists contains articles on many of the points above.

Software and code

Policy information about availability of computer code

Data collection	Freesurfer was used to reconstruct the cortical surfaces. Different versions of Freesurfer were used (documented in detail in Suppl Text F), as the cortical surfaces were sometimes provided by open databases (such as HCP).
Data analysis	Custom code was used in this study to derive various measures from a cortical surface. The code will be released together with the data (on Zenodo) upon acceptance of the manuscript.

For manuscripts utilizing custom algorithms or software that are central to the research but not yet described in published literature, software must be made available to editors/reviewers. We strongly encourage code deposition in a community repository (e.g. GitHub). See the Nature Research guidelines for submitting code & software for further information.

Data

Policy information about availability of data

All manuscripts must include a data availability statement. This statement should provide the following information, where applicable:

- Accession codes, unique identifiers, or web links for publicly available datasets
- A list of figures that have associated raw data
- A description of any restrictions on data availability

Full data (of all surface area, thickness, and curvature values in subjects) and analysis code will be made available upon acceptance of the manuscript on zenodo (link).

Field-specific reporting

Please select the one below that is the best fit for your research. If you are not sure, read the appropriate sections before making your selection.

☒ Life sciences ☐ Behavioural & social sciences ☐ Ecological, evolutionary & environmental sciences

For a reference copy of the document with all sections, see nature.com/documents/nr-reporting-summary-flat.pdf

Life sciences study design

All studies must disclose on these points even when the disclosure is negative.

Sample size	Sample sizes were not determined by a power calculation, but driven by data availability in large public databases.
Data exclusions	Data from the databases (subjects) were only excluded if their neuroimaging reconstruction in Freesurfer failed.
Replication	We use three public databases using healthy human subjects to verify our results.
Randomization	N/A
Blinding	N/A

Reporting for specific materials, systems and methods

We require information from authors about some types of materials, experimental systems and methods used in many studies. Here, indicate whether each material, system or method listed is relevant to your study. If you are not sure if a list item applies to your research, read the appropriate section before selecting a response.

Materials & experimental systems

n/a	Involved in the study	
☒	☐	Antibodies
☒	☐	Eukaryotic cell lines
☒	☐	Palaeontology
☒	☐	Animals and other organisms
☒	☐	Human research participants
☒	☐	Clinical data

Methods

n/a	Involved in the study	
☒	☐	ChIP-seq
☒	☐	Flow cytometry
☐	☒	MRI-based neuroimaging

Magnetic resonance imaging

Experimental design

Design type	Only structural (T1w) neuroimages used. We were only interested in the morphology of the brain
Design specifications	N/A
Behavioral performance measures	N/A

Acquisition

Imaging type(s)	structural
Field strength	varies between 1.5 and 3T depending on dataset, see Suppl Table F1 for details
Sequence & imaging parameters	Varies depending on dataset, see Suppl Table F1 for details
Area of acquisition	Whole cortex

Diffusion MRI ☐ Used ☒ Not used

Preprocessing

Preprocessing software	If not specified, we use the Freesurfer standard recon-all setting. However, some brain surfaces were provided by the database. Details can be found in Suppl Table F1

Normalization	Data were normalised using standard Freesurfer tools as part of the recon-all processing. See https://surfer.nmr.mgh.harvard.edu/fswiki/mri_normalize
Normalization template	Subject space
Noise and artifact removal	Bias field correction was performed using standard freesurfer tools as part of the recon-all pipeline
Volume censoring	No specific ROI/volume has been censored.

Statistical modeling & inference

Model type and settings	Various different models have been applied, described in Methods.
Effect(s) tested	No task/stimulus condition used

Specify type of analysis: ☐ Whole brain ☐ ROI-based ☒ Both

Anatomical location(s): Freesurfer's Desikan-Killiany Atlas

Statistic type for inference (See Eklund et al. 2016)	N/A
Correction	Specific to parts of the different analyses, specified where needed.

Models & analysis

n/a	Involved in the study
☒	☐ Functional and/or effective connectivity
☒	☐ Graph analysis
☒	☐ Multivariate modeling or predictive analysis
